# Emerging Variants of Canine Enteric Coronavirus Associated with Outbreaks of Gastroenteric Disease

**DOI:** 10.3201/eid3006.231184

**Published:** 2024-06

**Authors:** Edward Cunningham-Oakes, Jack Pilgrim, Alistair C. Darby, Charlotte Appleton, Chris Jewell, Barry Rowlingson, Carmen Tamayo Cuartero, Richard Newton, Fernando Sánchez-Vizcaíno, Ivo Salgueiro Fins, Bethaney Brant, Shirley Smith, Rebekah Penrice-Randal, Simon R. Clegg, Ashley P.E. Roberts, Stefan H. Millson, Gina L. Pinchbeck, P.-J.M. Noble, Alan D. Radford

**Affiliations:** University of Liverpool, Liverpool, UK (E. Cunningham-Oakes, J. Pilgrim, A.C. Darby, I.S. Fins, B. Brant, S. Smith, R. Penrice-Randal, G.L. Pinchbeck, P.-J.M. Noble, A.D. Radford);; Lancaster University, Lancaster, UK (C. Appleton, C. Jewell, B. Rowlingson);; University of Bristol, Bristol, UK (C.T. Cuartero, F. Sánchez-Vizcaíno);; University of Cambridge, Cambridge, UK (R. Newton);; University of Lincoln, Lincoln, UK (S.R. Clegg, A.P.E. Roberts, S.H. Millson)

**Keywords:** canine coronavirus, variants, evolution, epidemiology, surveillance, outbreak, seasonality, vomiting, viruses, zoonoses, enteric infections, United Kingdom

## Abstract

A 2022 canine gastroenteritis outbreak in the United Kingdom was associated with circulation of a new canine enteric coronavirus closely related to a 2020 variant with an additional spike gene recombination. The variants are unrelated to canine enteric coronavirus–like viruses associated with human disease but represent a model for coronavirus population adaptation.

Recent spillover events by coronaviruses highlights the potential devastating effects of emergence into human populations (*1*). Subsequent evolution can create variants in response to natural and vaccine-induced immunity. Canine enteric coronavirus (CECoV) is an alphacoronavirus with a complex evolutionary history punctuated by recombination (*2*). Type I and II CECoVs were largely defined by serologic differences; type I CECoVs also contain an additional open reading frame (*3*). Recently, type IIb and IIc (also called type I/II) variants were defined on the basis of recombination in the N terminal domain of the spike protein between type IIa CECoVs and either transmissible gastroenteritis virus of pigs or type I CECoV (*2*).

CECoV is generally associated with mild endemic canine gastroenteritis, and only sporadic reports of severe disease occur. Severe disease is usually associated with co-infection with other pathogens, known as pantropic CECoV (*4*). In 2020, an outbreak of gastroenteric disease occurred in dogs in the United Kingdom and was associated with a nationally distributed variant of CECoV (*5*). In 2022, a similar outbreak was reported on social media and initially affected coastal regions of Yorkshire, UK. Speculation about etiologies included contact with dead marine animals.

## The Study

We obtained electronic health data from the Small Animal Veterinary Surveillance Network (SAVSNET) (https://www.liverpool.ac.uk/savsnet). Veterinary data are collected passively by SAVSNET from ≈10% of veterinary practices in the United Kingdom, including a practitioner-recorded main presenting complaint (MPC). Laboratory data are collected passively by SAVSNET from participating diagnostic laboratories used by ≈60% of veterinary practices in the United Kingdom. We provided validated questionnaires to owners and veterinary surgeons to collect more detailed descriptive information. We recruited participants by using SAVSNET websites and social media.

We obtained canine fecal samples from 2 sources. We asked veterinary surgeons completing questionnaires to submit samples from dogs with vomiting, diarrhea, or both of unknown etiology. We also asked them to submit samples from control dogs. We additionally retrieved samples sent directly to IDEXX Laboratories (https://www.idexx.com) for testing for canine enteric pathogens after the completion of diagnostic testing (*6*). This study was approved by Liverpool University’s Central Committee (approval no. RETH00964) and Veterinary Research Ethics Committee (approval no. VREC922ab).

We modeled the weekly prevalence of gastroenteric MPC cases by using a logistic latent Gaussian process model, as done in previous studies (*5*), while adjusting for COVID-19–related disturbances in consult numbers. The modeling allowed us to capture the normal pattern of seasonal incidence in MPC cases; we considered the detection of high case prevalence as extreme relative to the model-based predictive distribution (Appendix).

We performed nucleic acid extraction, PCR, matrix gene sequencing, and analyses, as previously described (*5*). The diversity of sampled CECoVs required 2 approaches for whole-genome sequencing: initial sequence-independent single-primer amplification (SISPA) (*7*) and amplicon-tiling by using overlapping primers on the basis of SISPA-derived sequences (*8*) (Appendix).

We assessed recombination by visualizing whole genome alignments by using SimPlot++ 1.3 (https://github.com/Stephane-S/Simplot_PlusPlus). We aligned draft CECoV genomes and the nearest GenBank matches by using MAFFT (https://mafft.cbrc.jp/alignment/software). We identified regions of recombination by using Gubbins 2.3.4 (https://github.com/nickjcroucher/gubbins) and Phandango 1.3.0 (https://bivi.co/visualisation/phandango). We then masked those regions by using BEDTools 2.31.0 (https://bedtools.readthedocs.io/en/latest/index.html) and removed excessive gaps by using Gblocks 0.91b (https://gensoft.pasteur.fr/docs/gblocks/0.91b) before performing phylogenetic reconstruction with ModelFinder IQTree (http://www.iqtree.org/ModelFinder) and EvolView 3 (https://www.evolgenius.info).

The canine gastroenteric MPC was seasonal, peaking in January and February at 4%–5% of consultations. In Yorkshire, a 2-week period in 2022 exceeded 99% prediction intervals, consistent with an outbreak. Laboratory results showed CECoV diagnosis peaking each winter, with >20% of submitted samples positive (Appendix Figure 3). We received 28 questionnaire responses from veterinarians (20 cases, 8 controls) and 438 from owners (cases). The primary clinical signs were vomiting, diarrhea, and inappetence. Most cases lasted 3–7 days (7 [35.0%] of vet responses and 170 [38.8%] of owner responses). In co-habiting dogs, 108 (59.3%) were also unwell, suggesting possible transmission. Twenty-five percent of veterinary-reported cases and controls indicated a recent beach visit. All groups showed similar diet profiles.

**Figure 1:**
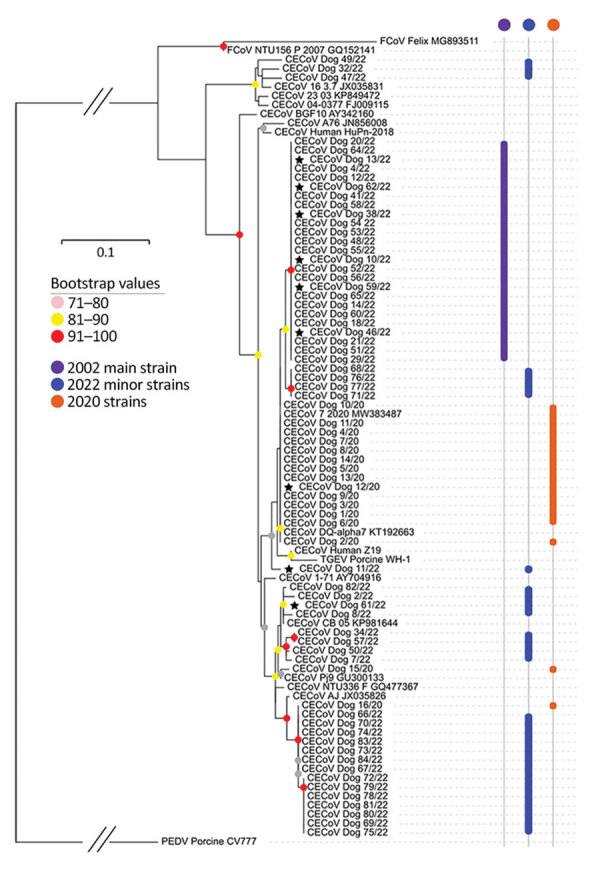
Maximum-likelihood tree of partial matrix gene (315-bp) sequences of canine enteric coronavirus recovered from infected canines, United Kingdom, 2020 and 2022. Sequences obtained from samples collected in 2022 are marked in purple (main strain) and blue (minor strains); stars indicate samples that were whole-genome sequenced as part of this study. Sequences obtained from 2020 are marked in orange. Scale bar indicates number of base differences per site.

We received 46 canine fecal samples from veterinary practices (45 cases, 1 control); 18 tested CECoV-positive. We obtained 87 samples from the diagnostic laboratory. We tested 27 and found that 19 were CECoV-positive, including 16 known CECoV-positive samples from the submitting laboratory. Matrix gene sequences for 36 of 37 amplicons were supplemented from a parallel study at the University of Lincoln. Phylogenetic analysis identified 1 main variant (25/55 sequences) (Figure 1) widely distributed across the United Kingdom (Appendix Figure 1); the remaining samples distributed into 14 minor variants.

**Figure 2:**
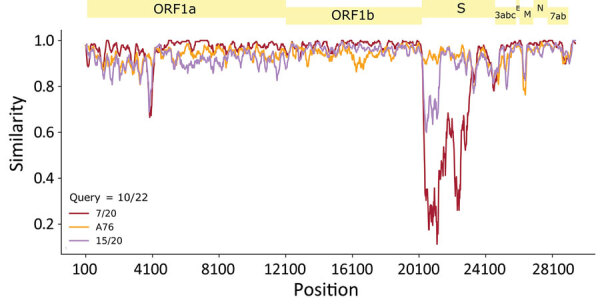
Viral sequences from 2022 identified from canine enteric coronavirus–infected canines in the United Kingdom, demonstrating a close relation to the 2020 major variant. The mismatched area was closely related to A76-type viruses, suggestive of a recombination event. Simplot analysis used the main variant observed in the 2022 United Kingdom outbreak (Dog 10/22) as a reference compared with the main (Dog 7/20) and minor (Dog 15/20) strains from the 2020 outbreak and the A76 strain. E, envelope; M, membrane; N, nucleocapsid; ORF, open reading frame; S, spike.

Our use of SISPA recovered 4 genomes, and by using primers on the basis of the sequence for Dog10/22, our amplicon tiling resulted in 6 additional near full-length genomes. Sequences of the 2022 major variant were most closely related to the 2020 major variant (Dog7/20) over most of the genome (96% coverage and 97.08% identity), and the gap in coverage was associated with low 5′ spike gene similarity (Figure 2). The mismatched area was closely related to A76-type viruses, suggestive of a recombination event. A core genome phylogeny excluding recombinant regions identified by Gubbins confirmed that the main 2022 variant was highly homogenous, clustering most closely with the main 2020 variant (Appendix Figure 2). All 2022 CECoVs from the United Kingdom were distinct from serotype IIb strains (Appendix Figure 4), which were associated with human pneumonia (HuPn-2018 and CECoV-Z19). The main 2020 variant, although classified as part of serotype IIb, lacked amino acid changes typically associated with a respiratory tropism (Appendix Figure 5).

## Conclusions

Our investigation showed a repeated winter rise in canine gastrointestinal disease nationally, which regionally exceeded normal seasonal prediction intervals and coincided with increased CECoV diagnoses. Questionnaire responses aided in refuting links to possible exposures to beaches and highlighted the severity and prolonged duration of many cases. Sequence analysis identified diverse variants of CECoV circulating in dogs in the United Kingdom during the 2022 sampling period, and 1 variant predominated. Whole-genome sequencing demonstrated that the predominant variant was closely related to a variant associated with a similar outbreak but had an additional spike gene recombination. We cannot formally link CECoV infection to disease; however, we suggest that because of the dominance of the 2022-sampled CECoV population by a single new variant, coupled with spike gene mutations likely to affect transmissibility or immunity (*2*), we may classify CECoV10/22 as a CECoV variant of interest in dogs (*9*).

Advances in sequencing technologies have enabled high-throughput and cost-effective methods to generate viral genomes for disease surveillance (*10*). Matrix gene PCR and SISPA of representative samples, followed by specific amplicon tiling, is an efficient strategy for surveillance in relatively resource-poor populations regardless of the affected species, where prior knowledge of circulating variants will likely be limited.

On the basis of resulting whole-genome sequences, our explanation for the origins of the predominant variant from 2022 is past co-infection with both a serotype I/II strain, such as an A76-type virus (*11*), and a virus like the major variant strain from 2020 (*2*). The complex mosaic nature of CECoV genomes suggests whole-genome sequencing is required for future surveillance. Because of the role of the spike protein in determining receptor binding, host affinity, immunoevasion, and severity of disease (*2*), the recombinant variants might behave differently from prototypical CECoV strains (*11*,*12*). Recent identification of CECoV variants in raccoon dogs (*Nyctereutes procyonoides*) from Wuhan, China, closely related to the major variant we identified in 2020 (*13*) and of CECoV-like viruses in humans (*14*,*15*) heightens the need for efficient surveillance of circulating CECoV variants in pet dogs and other domesticated species, wildlife, and humans.

Appendix Additional information about emerging variants of canine enteric coronavirus associated with outbreaks of gastroenteric disease.
