# Supplementary material for: Emerging Variants of Canine Enteric Coronavirus Associated with Outbreaks of Gastroenteric Disease
Source: Emerg Infect Dis. 2024 Jun;30(6):1240–4. doi: 10.3201/eid3006.231184 (PMC11139001; doi:10.3201/eid3006.231184)
Supplement: Appendix — Additional information about emerging variants of canine enteric coronavirus associated with outbreaks of gastroenteric disease. [file 23-1184-Techapp-s1.pdf]

Article DOI: https://doi.org/10.3201/eid3006.231184

EID cannot ensure accessibility for supplementary materials supplied by authors. Readers who have difficulty accessing supplementary content should contact the authors for assistance.

Emerging Variants of Canine Enteric Coronavirus Associated with Outbreaks of Gastroenteric Disease

Appendix

Supplementary methods

Sequence-Independent Single Primer Amplification (SISPA)

Briefly, 5μL TNA was incubated at 65°C for 5 minutes with 40pmol primer Sol-A (5′—GTT TCC CAC TGG AGG ATA NNN NN—3′) and 1 μL 10mM dNTP Mix before reverse transcription using SuperScript IV Reverse transcription (Thermo Fisher Scientific) following manufacturer's instructions with the addition of the ribonuclease inhibitor RNaseOUT (Life Technologies). Sequenase Version 2.0 (Thermo Fisher Scientific) was then used for second strand synthesis. cDNA was cleaned using AMPure XP beads in a 1.8:1 ratio and eluted in 40μL nuclease-free water. Random amplification was performed on each sample using Q5 High-Fidelity 2X Master Mix (New England Biosciences) and 100pmol primer Sol-B (5′—GTT TCC CAC TGG AGG ATA —3′). A PCR assay was then undertaken using the following conditions: initial denaturation for 30 seconds at 98°C, followed by 35 cycles of 98°C for 10 seconds, 54°C for 30 seconds, and 72°C for 1 minute, with a final extension step of 72°C for 10 minutes. Amplicons were then purified using AMPure XP beads in a 1.8:1 ratio and eluted in 40μL nuclease-free water. Amplified cDNA was quantified using a Qubit 1X dsDNA HS hit and Qubit Flex Fluorometer (Thermo Fisher Scientific) with fragment lengths assessed using the Agilent 2100 Bioanalyzer system and High Sensitivity DNA Kit (Agilent). Library preparation was performed as previously described by Gauthier et al. (*1*) [see end of this file]. Five samples were multiplexed on each MinION flow cell, with the addition of a negative control (Nuclease-free water) to each library. Samples were sequenced on FLO-MIN106D flow cells (R9.4.1 chemistry)

on a GridION sequencing device for 72 hours using MinKNOW (Version 3.6.5) with live base calling disabled.

Amplicon tiling

The version 2 primer scheme which was based on a preliminary SISPA genome, were as described by Quick et al. (*2*). For details of primer schemes, reference genomes and version 1 (pilot) data, see https://github.com/edwardcunningham-oakes/CECoV-outbreak-2022. Library preparation of targeted PCR amplicons was adapted from the ncov-2019 sequencing v3 (ARTIC) protocol by Josh Quick (https://www.protocols.io/view/ncov-2019-sequencing-protocol-v3-locost-bh42j8ye) with slight modifications as follows: PCR product (amplicon size 1200bp) was normalized to 100ng per sample for end-preparation and adaptor ligation steps. The ONT Native Barcoding Ligation kit (EXP-NBD196) was used for multiplexing no more than 30 samples per flow cell. All samples were sequenced on FLO-MIN106D flow cells (R9.4.1 chemistry) on a GridION sequencing device for 72 hours using MinKNOW (Version 3.6.5) with live basecalling disabled.

Bioinformatics

Basecalling of SISPA and amplicon tiling Fast5 files was undertaken using Guppy v4.2.2. Outputted FASTQ files were demultiplexed using PoreChop v0.2.4 (*3*) and quality filtered/primer trimmed using Nanofilt version 2.8.0 (size selection: 150–1500bp (SISPA), 1000–1400bp (amplicon-tiling); Average Q score: ≥15; Head and tail trimming: 18 bases (SISPA), 27 bases (amplicon-tiling)) (*4*). Filtered and trimmed SISPA FASTQ files were uploaded to the online BugSeq v1 portal https://bugseq.com upload date: Mar 29, 2022) for metagenomic classification using the RefSeq database (*5*) with classification results summarized and viewed in Recentrifuge (*6*). Reads classified as alphacoronavirus were extracted using seqtk version 1.3-r106 (https://github.com/lh3/seqtk). BLASTn was then used on a subset of the SISPA and amplicon-tiling reads to find the nearest Genbank hit to use as a reference for mapping reads to build a consensus. The mpileup option in Samtools version 1.15 (*7*) was used to build a consensus with areas of less than 5X coverage masked. For one genome (Dog 61/22) which had several low coverage areas (<5X depth), amplicons from the V1 amplicon tiling scheme were incorporated to generate a consensus sequence. If reference genomes were not sufficient for mapping the divergent Spike gene, a custom BLAST database of canine

coronaviruses spike genes was used to map reads to get a consensus of the S gene to be combined with the draft genome.

Gubbins was used to assess recombination, based on genome alignments and phylogenetic relationships. Single nucleotide polymorphisms (SNPs) were identifed in aligned whole-genome sequence data of related genomes, and used to constructs an initial phylogenetic tree (RAxML. GTRCAT as the model). Regions of putative recombination were defined by using a minimum threshold of 3 SNPs (default).

In addition, and as alphacoronavirus spike genes have been shown to undergo intragenic recombination, phylogenies of the different domains of S genes generated in this study and other alphacoronaviruses (Feline/Canine coronaviruses I and II and Transmissible gastroenteritis virus) were generated using the above methods with slight modifications as follows: Pal2NAL (*8*) was used to ensure accurate codon alignments and the GTR+F+G4 model was used for tree construction.

Temporal analysis of Major Presenting Complaint (MPC) data

During our period of interest, the presence of social distancing (lockdown) restrictions due to COVID-19 had a marked effect on the apparent prevalence of MPCs. The cancellation of routine consults (e.g., vaccinations and health checks) reduced N_t for weeks during which social distancing restrictions applied, though emergency consults for gastroenteric disease appear to still have taken place (*9*). The result of this was to increase the apparent prevalence of MPC during the affected weeks. To capture this effect, we introduced a dummy variable, z_t taking the value 1 if week t was affected by social distancing and 0 otherwise (*10*).

We then let

$$\log\left(\frac{p_t}{1-p_t}\right) = \alpha + \beta t + \delta z_t + u_t$$

where α is the mean log odds of an MPC consult, β represents a linear time trend capturing long-term drift in MPC prevalence (the effect on the log odds of MPC for a 1 week increase in time), δ represents an offset in the log odds for MPC for weeks in which social distancing was imposed, and u_t represents a time-varying random effect.

The random effect u_t allows us to model periodic serial correlation in our weekly observations, as well as any extra-Binomial variation that might contribute to the overall variability of cases from 1 week to the next. We model the vector $\boldsymbol{u}$ as a Gaussian process with mean 0 and covariance matrix Σ^2 such that

$$\mathbf{u} \sim \mathrm{MultivariateNormal}(\mathbf{0}, \Sigma^2).$$

The covariance matrix Σ^2 captures the correlation between two variates u_t and u_s spaced s-t weeks apart, and we assume the correlation follows a periodic function

$$\Sigma^2_{ts} = \begin{cases} \sigma^2 + \tau^2 & \text{if } s = t \\ \sigma^2 \exp\left(-\dfrac{\sin^2\left(\pi |s-t| \frac{7}{365}\right)}{2\phi^2} \right) & \text{otherwise} \end{cases}$$

Here, σ^2 represents the variance between two timepoints spaced a year apart, ϕ represents the lengthscale of the correlation (essentially how correlated any two adjacent timepoints are), and τ^2 represents extra-Binomial variability due to observation error. For identifiability reasons, we fix $\phi = 0.32\text{year}^{-1}$, tuned manually to give a satisfactory amount of smoothing over the timeseries.

The model was cast in a Bayesian setting, for which prior distributions were used for all unknown parameters α, β, δ, σ, τ (Table 1).

The model was fitted using the No-U Turn Sampler implementation in the Python package PyMC3 for 6000 iterations with a 1000 iteration burn in period, to provide numerical estimates of the joint posterior distribution (*11*).

The advantage of Bayesian inference in our context is that it allows us to compare our observations y_t, $t = 1,...,174$, with the filter distribution

$$\pi(y'_t|\mathbf{y}) = \int_\Theta f(y'_t|\theta)\pi(\theta|\mathbf{y})\mathrm{d}\theta$$

For each observation, we calculate

$$q_t = \Pr(y_t > y'_t)$$

highlighting timepoints where $0.95<q_t<0.99$ as possibly higher than expected, and where $q_t>0.99$ as likely to be higher than expected. Conversely, we identify cases where $0.025 <q_t <0.05$ and $q_t <0.025$ as possibly or likely to be lower than expected respectively.

References

1. Gauthier NPG, Nelson C, Bonsall MB, Locher K, Charles M, MacDonald C, et al. Nanopore metagenomic sequencing for detection and characterization of SARS-CoV-2 in clinical samples. PLoS One. 2021;16:e0259712. PubMed https://doi.org/10.1371/journal.pone.0259712

2. Quick J, Grubaugh ND, Pullan ST, Claro IM, Smith AD, Gangavarapu K, et al. Multiplex PCR method for MinION and Illumina sequencing of Zika and other virus genomes directly from clinical samples. Nat Protoc. 2017;12:1261–76. PubMed https://doi.org/10.1038/nprot.2017.066

3. Wick RR, Judd LM, Gorrie CL, Holt KE. Completing bacterial genome assemblies with multiplex MinION sequencing. Microb Genom. 2017;3:e000132. PubMed https://doi.org/10.1099/mgen.0.000132

4. De Coster W, D'Hert S, Schultz DT, Cruts M, Van Broeckhoven C. NanoPack: visualizing and processing long-read sequencing data. Bioinformatics. 2018;34:2666–9. PubMed https://doi.org/10.1093/bioinformatics/bty149

5. Fan J, Huang S, Chorlton SD. BugSeq: a highly accurate cloud platform for long-read metagenomic analyses. BMC Bioinformatics. 2021;22:160. PubMed https://doi.org/10.1186/s12859-021-04089-5

6. Martí JM. Recentrifuge: Robust comparative analysis and contamination removal for metagenomics. PLOS Comput Biol. 2019;15:e1006967. PubMed https://doi.org/10.1371/journal.pcbi.1006967

7. Li H, Handsaker B, Wysoker A, Fennell T, Ruan J, Homer N, et al., 1000 Genome Project Data Processing Subgroup. The sequence alignment/map format and SAMtools. Bioinformatics. 2009;25:2078–9. PubMed https://doi.org/10.1093/bioinformatics/btp352

8. Suyama M, Torrents D, Bork P. PAL2NAL: robust conversion of protein sequence alignments into the corresponding codon alignments. Nucleic Acids Res. 2006;34:W609-12. PubMed https://doi.org/10.1093/nar/gkl315

9. Littlehales R, Noble PM, Singleton DA, Pinchbeck GL, Radford AD. Impact of Covid-19 on veterinary care. Vet Rec. 2020;186:650–1. PubMed https://doi.org/10.1136/vr.m2495

10. The Institute for Government. Timeline of UK government coronavirus lockdowns and restrictions. 2022 [cited 2022 Aug 28]. https://www.instituteforgovernment.org.uk/charts/uk-government-coronavirus-lockdowns

11. Salvatier J, Wiecki TV, Fonnesbeck C. Probabilistic programming in Python using PyMC3. PeerJ Comp. Sci. 2016;2:e55. https://doi.org/10.7717/peerj-cs.55

Appendix Table. Prior distributions used for the longitudinal latent Gaussian process model

Parameter	Prior Distribution
α	Normal (0,1000)
$\boldsymbol{\beta}$	Normal (0,100)
σ^2	Half Normal (5)
τ^2	Half Normal (5)
δ	Normal (0,100)

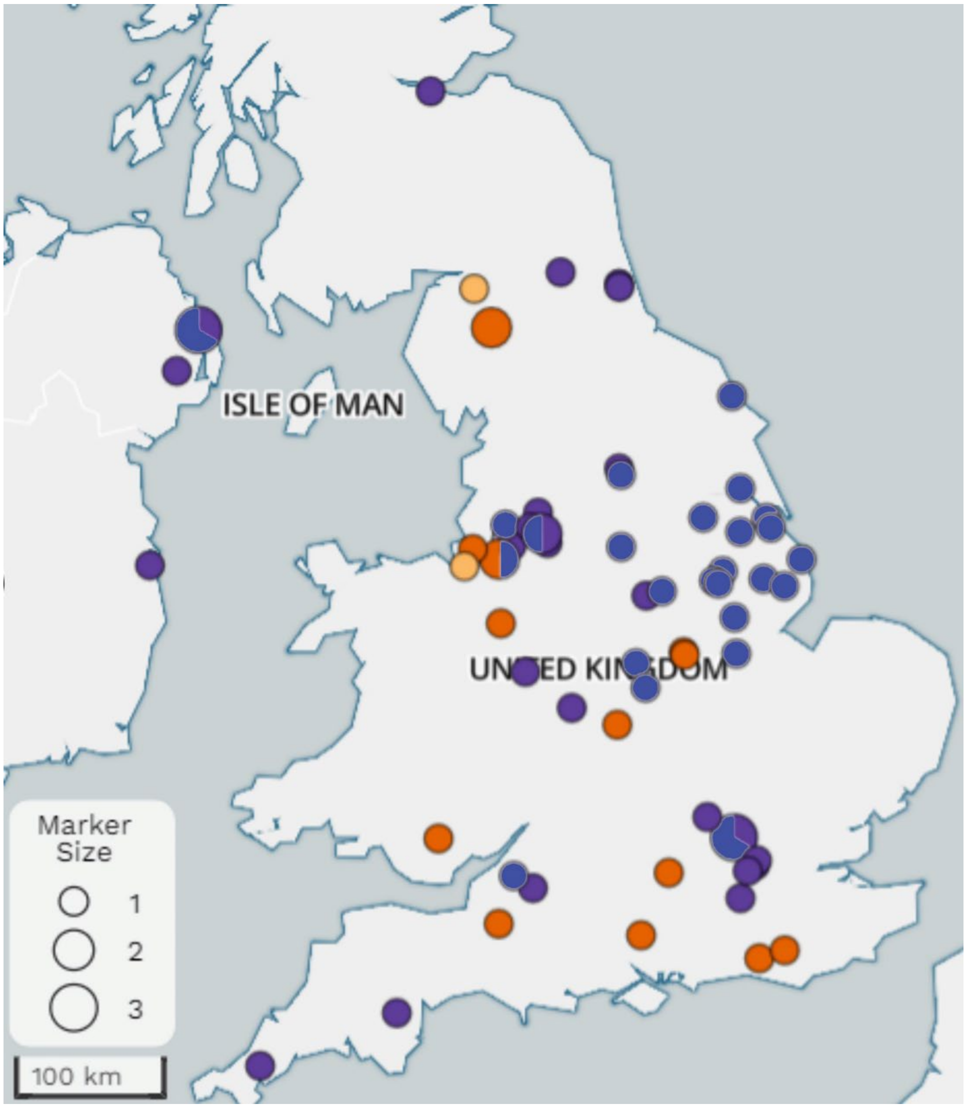

Appendix Figure 1. Sequences and location mapping of the canine enteric coronavirus viral sequences recovered from infected canines, United Kingdom, 2020 and 2022. Sequences obtained from samples collected in this study (2022) are marked in purple (main strain) and blue (minor strains). Sequences obtained from 2020 are marked in orange. Geographic distribution of canine enteric coronavirus matrix gene variants across the United Kingdom. Each location identifier can be linked to the phylogeny by using an interactive interface (available at https://microreact.org/project/uZuCTLwtXkNQxpodQeZFS6-cecov-m-gene-2022).

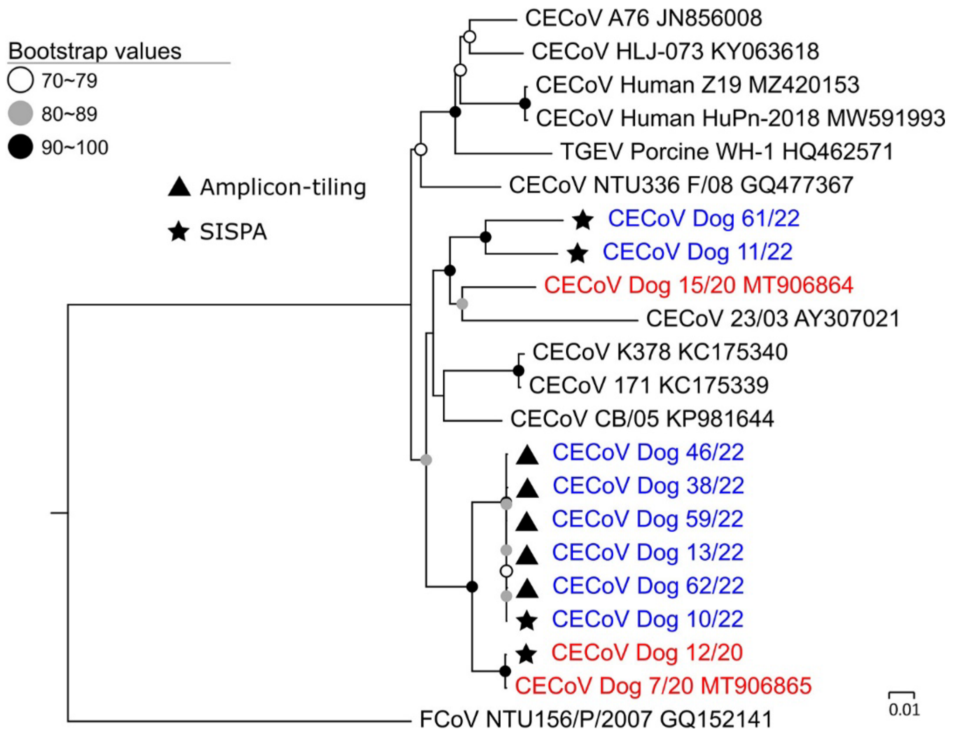

Appendix Figure 2. Viral sequences from 2022 identified from canine enteric coronavirus (CeCoV)-infected canines in the United Kingdom demonstrated a close relation to the 2020 CeCov major variant. The mismatched area was closely related to A76-type viruses, suggestive of a recombination event. Maximum-likelihood phylogenomic analysis (final alignment 23,632 bp) of CECoV core genomes from 2020 (red) and 2022 (blue) United Kingdom outbreaks alongside the closest matching GenBank matches. Sequences identified in this study are indicated by stars (sequence-independent single-primer amplification) and triangles (amplicon-tiling). The spike protein gene and other areas of recombination, as detected by Gubbins, were excluded from analysis. E, envelope; M, membrane; N, nucleocapsid; S, spike.

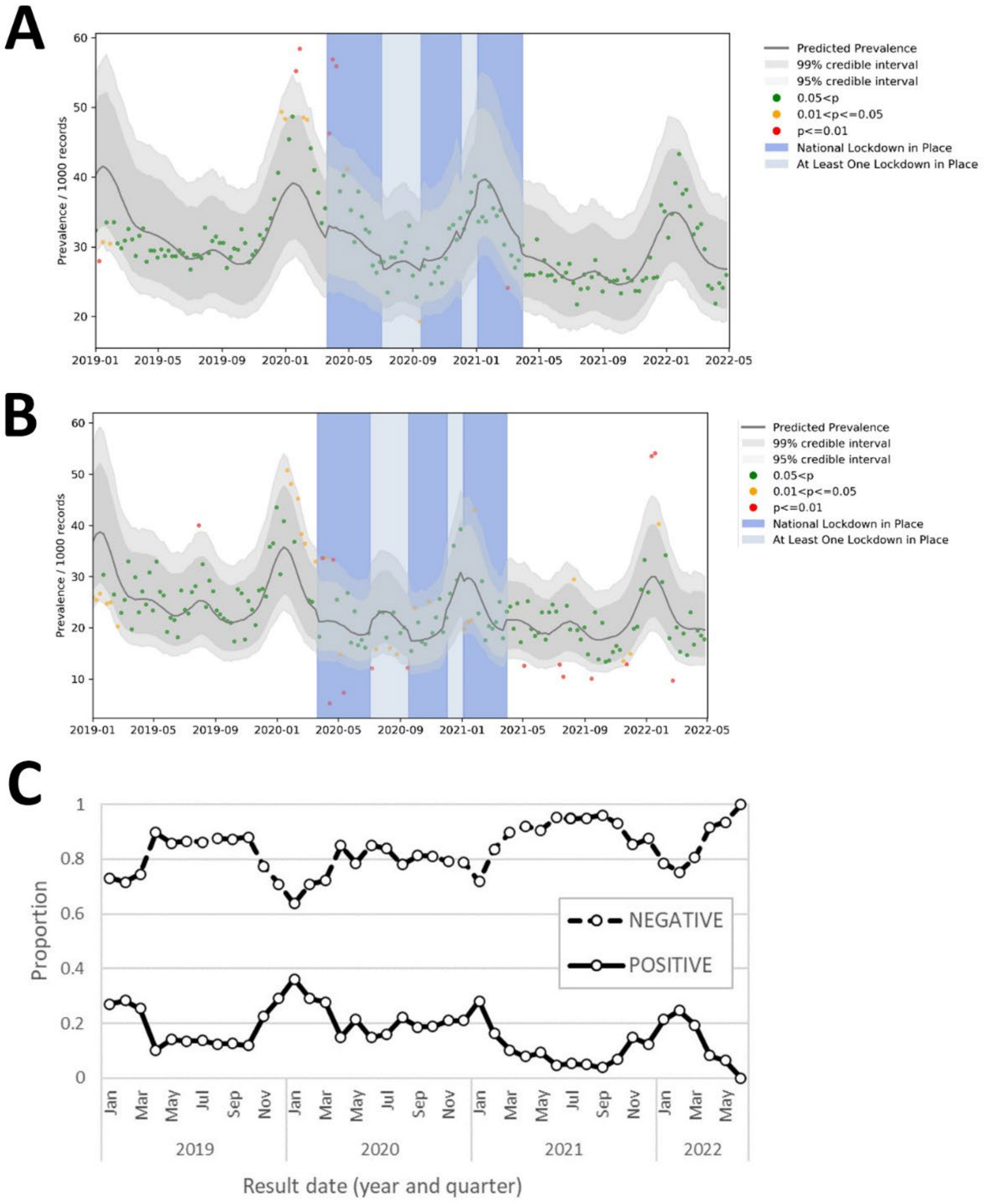

Appendix Figure 3. Yorkshire (A) and National (B) temporal distributions of consultations classified by the attending practitioner as mainly associated with gastroenteric disease. Approximate times of UK COVID lockdowns are indicated; lockdowns had a profound impact on the cases seen by practitioners (data not presented) but were not associated with the observed changing patterns of gastroenteric disease. C) Temporal distribution of the proportion of CECoV samples testing positive and negative from diagnostic laboratories.

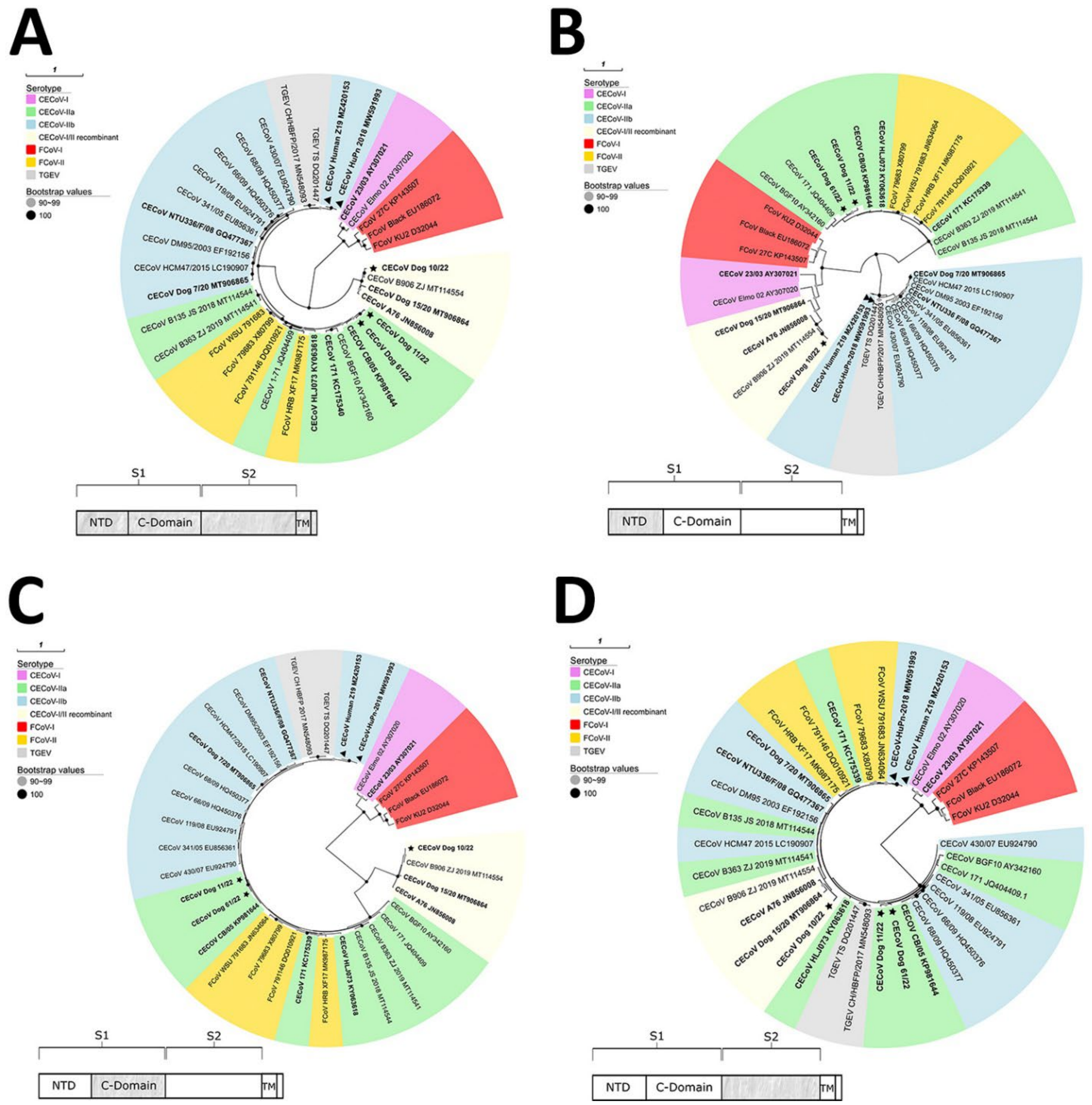

Appendix Figure 4. Panel of S-gene maximum-likelihood phylogenies for serotype I/II canine enteric coronaviruses (CECoV), feline coronaviruses (FCoV) and transmissible gastroenteritis viruses (TGEV) as well as recombinant strains. A) Full spike gene B) N terminal domain (NTD) of S1 region C) C-domain of S1 region D) S2 region. Stars indicate 2022 UK strains. Triangles indicate human pneumonia strains. TM = transmembrane domain

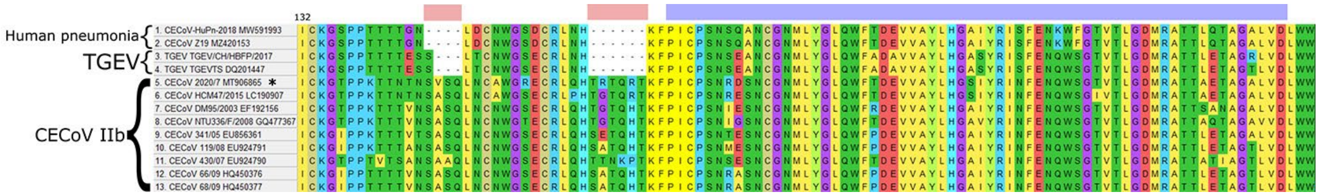

Appendix Figure 5. Amino acid sequence alignment of the S1 NTD region (modified from Zehr et al. (2022). Viruses 14, 853). The blue bar represents the area identified experimentally as being pertinent to sialic acid binding (a viral tropism determinant) in TGEV. The red bars represent upstream deletions which could potentially impact sialic acid binding affinity. These changes have been suggested to lead to a loosening of an enteric tropism, potentially contributing to the respiratory tropism observed in both human pneumonia and TGEV alphacoronaviruses. Asterisk indicates the major UK 2020 variant previously identified by the authors (*5*). The number at the beginning of the alignment indicates amino acid position.
